# Aging-related biomarkers for the diagnosis of Parkinson’s disease based on bioinformatics analysis and machine learning

**DOI:** 10.18632/aging.205954

**Published:** 2024-09-10

**Authors:** Weiwei Yang, Shengli Xu, Ming Zhou, Piu Chan

**Affiliations:** 1 Department of Neurobiology, Neurology and Geriatrics, Xuanwu Hospital of Capital Medical University, National Clinical Research Center for Geriatric Disorders, Beijing, China; 2 Clinical Center for Parkinson's Disease, Capital Medical University, Beijing, China; 3 Key Laboratory for Neurodegenerative Disease of the Ministry of Education, Beijing Key Laboratory for Parkinson's Disease, Parkinson Disease Center of Beijing Institute for Brain Disorders, Beijing, China

**Keywords:** Parkinson’s disease, aging, nomogram, machine learning algorithms, diagnostic biomarker

## Abstract

Parkinson’s disease (PD) is a multifactorial disease that lacks reliable biomarkers for its diagnosis. It is now clear that aging is the greatest risk factor for developing PD. Therefore, it is necessary to identify novel biomarkers associated with aging in PD. In this study, we downloaded aging-related genes from the Human Ageing Gene Database. To screen and verify biomarkers for PD, we used whole-blood RNA-Seq data from 11 PD patients and 13 healthy control (HC) subjects as a training dataset and three datasets retrieved from the Gene Expression Omnibus (GEO) database as validation datasets. Using the limma package in R, 1435 differentially expressed genes (DEGs) were found in the training dataset. Of these genes, 29 genes were found to occur in both DEGs and 307 aging-related genes. By using machine learning algorithms (LASSO, RF, SVM, and RR), Venn diagrams, and LASSO regression, four of these genes were determined to be potential PD biomarkers; these were further validated in external validation datasets and by qRT-PCR in the peripheral blood mononuclear cells (PBMCs) of 10 PD patients and 10 HC subjects. Based on the biomarkers, a diagnostic model was developed that had reliable predictive ability for PD. Two of the identified biomarkers demonstrated a meaningful correlation with immune cell infiltration status in the PD patients and HC subjects. In conclusion, four aging-related genes were identified as robust diagnostic biomarkers and may serve as potential targets for PD therapeutics.

## INTRODUCTION

Parkinson’s disease (PD) is a common neurodegenerative disease in older adults and characterized by a range of clinical manifestations. These include motor symptoms like resting tremor, bradykinesia, myotonia, and postural balance disorder, besides non-motor symptoms such as sleep disturbances, olfactory dysfunction, gastrointestinal issues, mood disorders, and cognitive impairment [1, 2]. Despite these well-recognized symptoms, PD is considered as a multifactorial disease, because of its complexity etiology [3, 4].

The diagnosis of Parkinson’s disease (PD) predominantly relies on clinical features, posing significant challenges for achieving precision in diagnosis. Despite extensive research directed towards the exploration of potential biomarkers such as neuroimaging, cerebrospinal fluid (CSF) analysis, serum, and saliva biomarkers, the quest for reliable biomarkers for PD diagnosis remains lacking [5–7]. Hence, the study of potential biomarkers and the elucidation of their molecular mechanisms stand as necessary things in advancing the diagnosis and treatment of Parkinson’s disease (PD).

Most studies on this topic have been on utilizing cerebrospinal fluid (CSF) samples from patients to assess biomarkers associated with Parkinson’s disease (PD) progression and severity [8]. However, the using of CSF-based biomarkers remains constrained due to the invasive nature of sample collection, necessitating skilled health worker, and posing risks. Consequently, exploring blood-based biomarkers presents a compelling alternative, offering advantages in terms of simplicity, reduced discomfort, and decreased risk. PD exhibits considerable heterogeneity, with only a minority of cases attributed to causal monogenetic mutations, while a majority are associated with multiple low-risk genetic loci, collectively contributing to sporadic PD [9]. Despite the modest effect size of individual loci, their cumulative impact substantially elevates PD risk [10]. Notably, these genetic variations primarily modulate gene expression rather than altering protein characteristics [11]. Both monogenetic and sporadic forms of PD manifest in the hallmark neuronal loss in the substantia nigra (SN) and the aggregation of misfolded α-synuclein in Lewy bodies. The age-related rise in PD incidence underscores the accrual of genetic alterations through dysregulated gene expression over time [12]. Traditionally conceptualized as a disorder affecting specific neuronal populations, PD poses challenges in procuring brain tissue for transcriptomic analyses. However, intriguingly, emerging evidence suggests that the gene expression profile in peripheral blood exhibits remarkable similarity to that of the brain [13, 14].

Therefore, differential expression analysis of genes (DEGs) in the peripheral blood of PD patients holds promise as ideal biomarkers—measuring them would be safe, less invasive, cost-effective, and expeditious, ensuring minimal discomfort for patients. Identifying DEGs in blood could serve as a valuable tool for elucidating the multifaceted molecular alterations characteristic of PD, potentially offering diagnostic and prognostic insights, as well as guiding therapeutic interventions. Thus, we sought to measure the whole-blood RNA levels of idiopathic PD patients and control subjects using high-throughput sequencing.

In recent years, the advent of high-throughput sequencing technologies has facilitated the identification of gene expression signatures, underlying biological processes, and potential therapeutic targets in Parkinson’s disease (PD). Notably, whole-genome sequencing was first employed in 2014 to comprehensively catalog all long noncoding RNAs (lncRNAs) present in PD patients, revealing aberrant expression patterns in at least 6000 distinct lncRNAs [15]. Bioinformatics methods can be used to efficiently analyze biological data, identifying candidate genes or gene sets relevant to the onset and progression of diseases, followed by experimental verification.

Aging stands as the primary risk factor for Parkinson’s disease (PD), implying that mechanisms driving the aging process likely contribute to PD neurodegeneration [16]. Multiple lines of evidence imply the association between aging and PD. Firstly, characteristic features of brain aging, including mitochondrial dysfunction, oxidative stress [17, 18], disruptions in protein homeostasis [19], and neuroinflammation [20], are intricately linked to the pathogenesis of PD. Secondly, mutations responsible for monogenic forms of PD may be present from conception but typically manifest clinically only after a period of aging [16, 21]. Lastly, interventions aimed at extending lifespan through genetic, dietary, or pharmacological means often demonstrate a protective effect against PD-related neurodegeneration [22]. These observations highlight the central role of aging in the development of PD and suggest that advancements in our understanding of the biology of aging could offer novel insights into the underlying mechanisms of PD pathophysiology.

In recent years, significant research progress has been made in identifying biomarkers associated with aging-related neurodegenerative disorders through comprehensive bioinformatics analyses [23–25]. However, the availability of diagnostic biomarkers for Parkinson’s disease (PD) based on aging-related genes remains limited. Concurrently, the rapid advancement of artificial intelligence has facilitated the utilization of machine learning algorithms, including Support Vector Machine (SVM), Least Absolute Shrinkage and Selection Operator (LASSO), Random Forest (RF), and Ridge Regression (RR), for the identification of key diagnostic biomarkers in neurodegenerative diseases.

Therefore, we use bioinformatic analyses and machine learning algorithms to identify potential diagnostic biomarkers for PD. Through this methodology, we pinpointed four aging-related genes with promising diagnostic utility in PD. Furthermore, we constructed molecular regulatory networks associated with these diagnostic biomarkers, thereby laying a foundation for elucidating the molecular mechanisms for PD pathophysiology.

## RESULTS

### DEGs involved in PD

**Figure 1:**
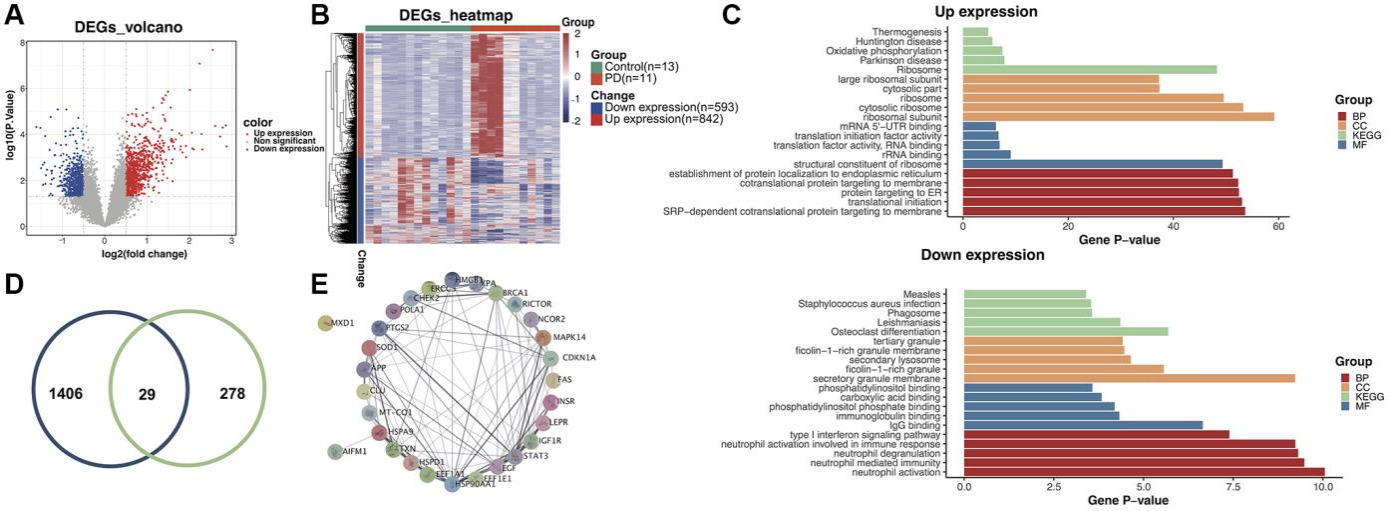
**Identification of aging-related differentially expressed genes (DEGs) in the training dataset.** (**A**) Volcano plot of the DEGs. (**B**) Heatmap of the DEGs. (**C**) Gene ontology and Kyoto Encyclopedia of Genes and Genomes (KEGG) enrichment analysis of overexpressed and underexpressed DEGs. (**D**) Intersection of aging-related genes and DEGs. (**E**) Protein-protein interaction (PPI) network analysis reveals that 28 genes interact with each other.

Using a cutoff threshold of |logFC| >1 and *p*-value < 0.05 for self-test RNA-Seq data from 11 PD patients and 13 HC subjects, a total of 1435 DEGs were identified in the training dataset. Among the DEGs, 842 genes were found to be upregulated and 593 genes downregulated in PD patients (Figure 1A). The expression levels of these DGEs were visualized using a heatmap (Figure 1B). To elucidate the biological function of these DEGs, Gene Ontology (GO) term enrichment analysis was conducted, including analysis for enriched BP, MF, and CC. Furthermore, KEGG pathway enrichment analysis was performed to delineate the potential involvement of these DEGs in specific biological pathways (Figure 1C). The KEGG results illustrated that the upregulated genes were mainly enriched in the thermogenesis, Huntington’s disease, oxidative phosphorylation, Parkinson’s disease, and ribosome pathways. However, the downregulated genes were enriched in the measles, *Staphylococcus aureus* infection, phagosome, leishmaniasis, and osteoclast differentiation pathways. Using the Human Ageing Gene Database, 307 aging-related genes were obtained. The overlap between DEGs from the RNA-Seq data and the aging-related genes were visualized in a Venn diagram (Figure 1D). Finally, 29 genes that were found in both sets were identified as potential diagnostic biomarkers. Using the STRING database, we studied the PPI network of above genes, and Cytoscape V3.9.1 was applied to visualize the network (Figure 1E). The MXD1 gene had no significant network connections and was thus excluded from subsequent studies.

### Identification and validation of optimal biomarkers in PD

**Figure 2:**
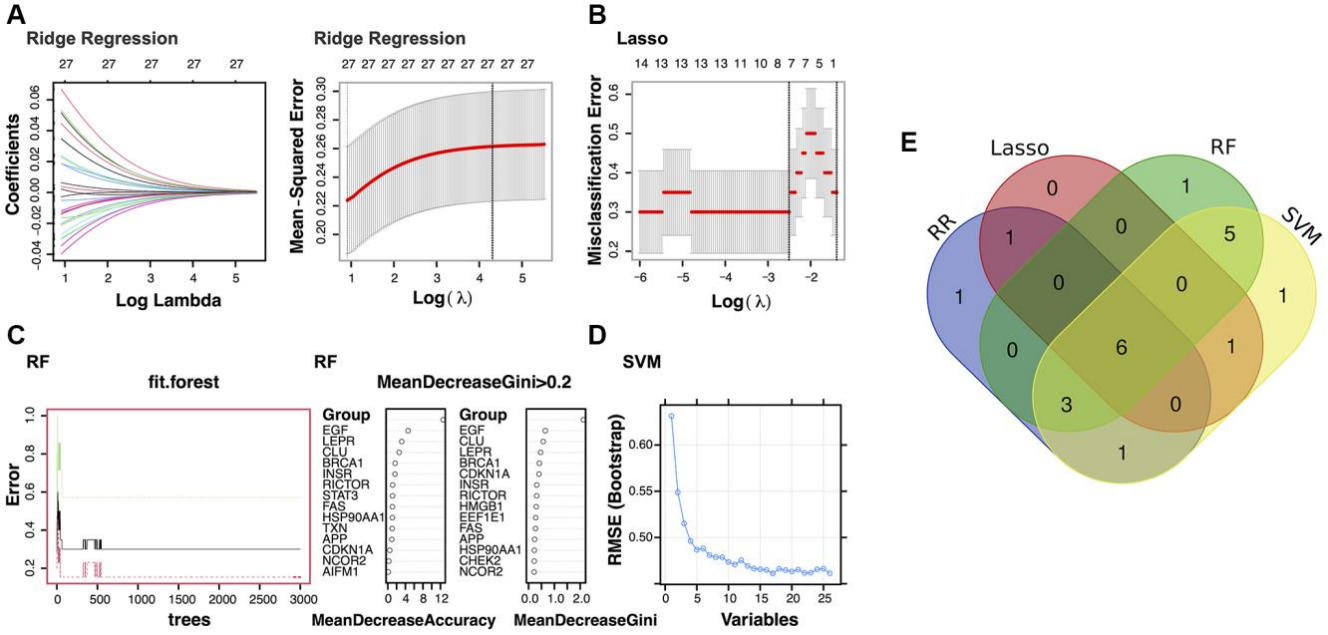
**Identification of the potential diagnostic biomarkers from the selected modules.** (**A**) Ridge Regression analysis (RR). (**B**) Least Absolute Shrinkage and Selection Operator (LASSO) regression analysis. (**C**) Random Forest (RF) analysis. (**D**) Support Vector Machine (SVM) analysis. (**E**) Venn plot exhibiting the biomarkers that were identified by all four algorithms.

**Figure 3:**
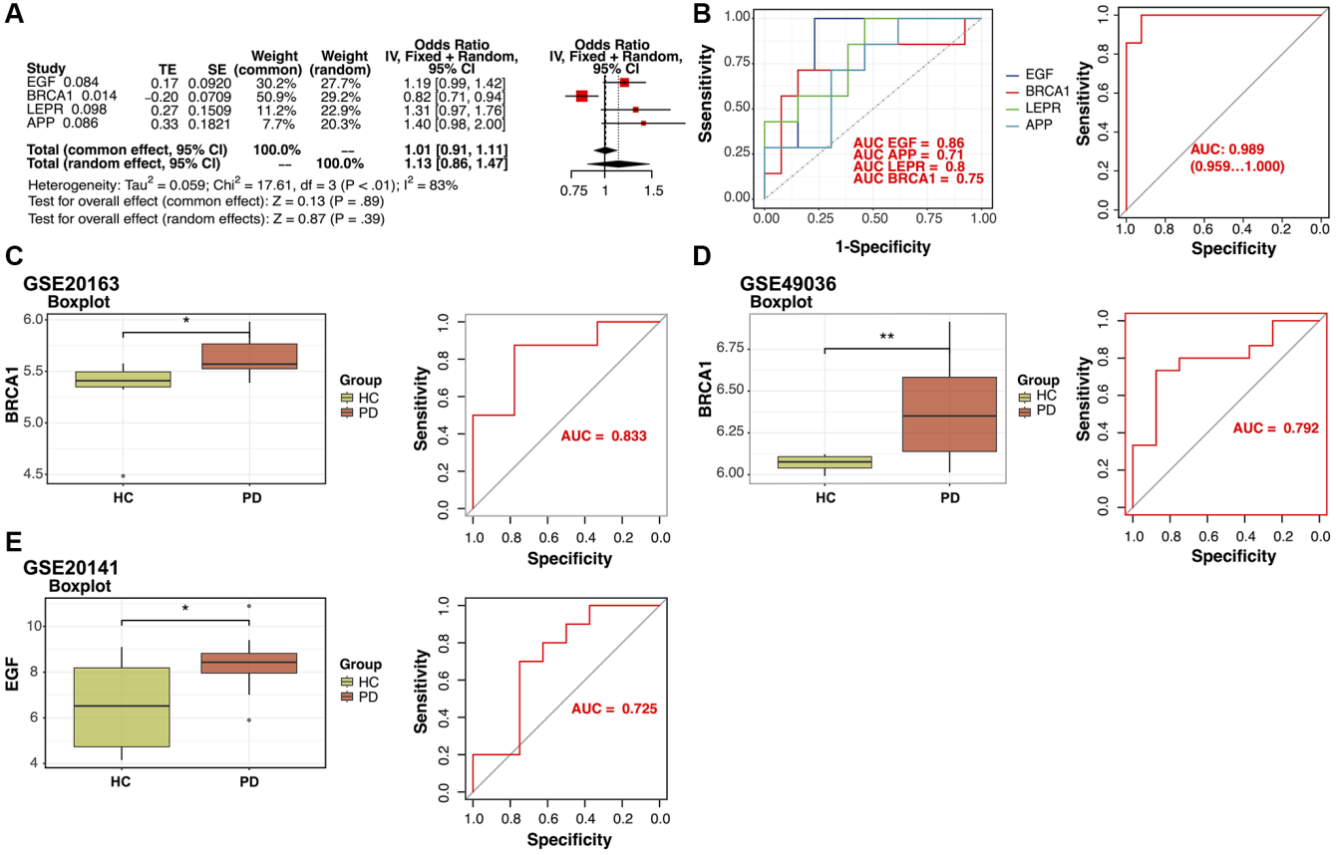
**Verification of the identified potential diagnostic biomarkers.** (**A**) Least Absolute Shrinkage and Selection Operator (LASSO) regression analysis identified four potential diagnostic biomarkers (*p*-value < 0.1). (**B**) Receiver operating characteristic (ROC) curves for evaluating the diagnostic ability of the four genes separately or combined in a training cohort. (**C**, **D**) Box plots for the differential expression analyses of BRCA1 in the validation datasets GSE20163 and GSE49036. ROC curves for evaluating the diagnostic ability of BRCA1 in the validation datasets. (**E**) Box plots for the differential expression analysis of EGF in the validation dataset GSE20141. ROC curves for evaluating the diagnostic ability of EGF in GSE20141.

In the study, 12 genes were identified as potential diagnostic biomarkers from the modules using the RR algorithm (Figure 2A). Additionally, using the LASSO regression algorithm, eight genes were identified from the selected modules as potential diagnostic biomarkers (Figure 2B). The RF algorithm identified 15 potential diagnostic biomarkers (Mean Decrease Gini >0.2; Figure 2C). Through the application of the SVM-RFE algorithm, 17 genes were identified as potential diagnostic biomarkers from the modules (Figure 2D). Subsequently, through a Venn diagram analysis, six genes (EGF, BRCA1, CLU, LEPR, CHEK2, and APP) were found to be overlapping and thus identified as robust diagnostic biomarkers (Figure 2E). Following this, dimensionality reduction via LASSO regression led to the identification of four genes for the construction of a diagnostic model for PD (EGF, BRCA1, LEPR, and APP; *p*-value < 0.1; Figure 3A, 3B).

The diagnostic performance of four potential diagnostic biomarkers was assessed through ROC curve analysis. The areas under the ROC curves for each gene were as follows: EGF (AUC = 0.86), APP (AUC = 0.71), LEPR (AUC = 0.8), and BRCA1 (AUC = 0.75). Upon combining all four potential diagnostic biomarker genes into a single diagnostic model, the AUC reached 0.98 in the training dataset. Subsequently, external validation of the diagnostic efficacy of these four genes was conducted using GSE20163, GSE49036, and GSE20141 datasets. The results revealed a notable difference in BRCA1 expression in the GSE20163, GSE49036 datasets (Figure 3C, 3D) and good diagnostic accuracy for the detection of PD: in GSE20163, AUC = 0.833 and in GSE49036, AUC = 0.792. In data from GSE20141, only EGF showed high expression in the PD group and good prediction efficacy (AUC = 0.725; Figure 3E).

### PPI network of four potential diagnostic biomarkers in PD and enrichment analysis

**Figure 4:**
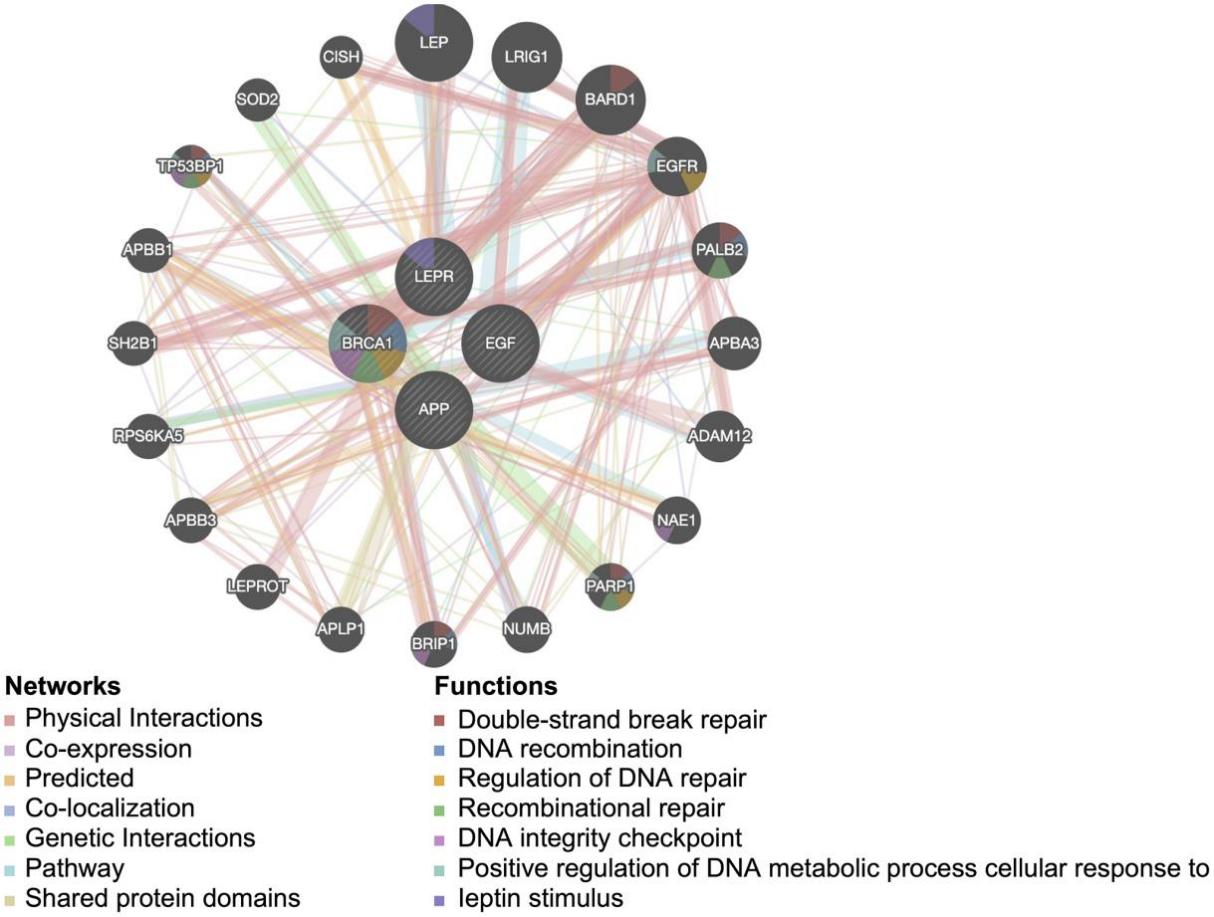
**Protein-protein interaction network for the four potential diagnostic biomarkers constructed in GeneMANIA.** Different colors of the network edge indicate the bioinformatics method applied: physical interaction, coexpression, predicted, colocalization, pathway, genetic interaction, and shared protein domains.

**Figure 5:**
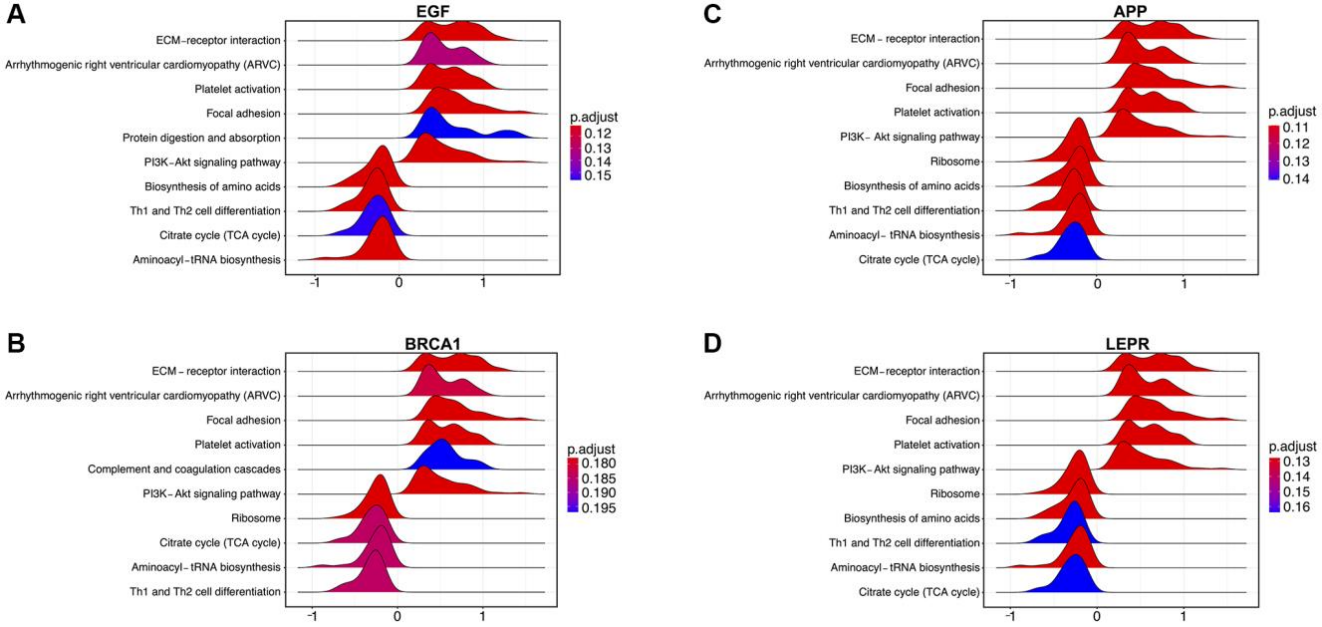
**Enrichment analysis of the four potential biomarker genes according to gene set enrichment analysis (GSEA) in the training dataset.** (**A**) Pathways enriched in EGF-mediated signaling, (**B**) BRCA1-mediated signaling, and (**C**) signaling by APP in Parkinson’s disease (PD). (**D**) Signaling by LEPR in PD.

**Figure 6:**
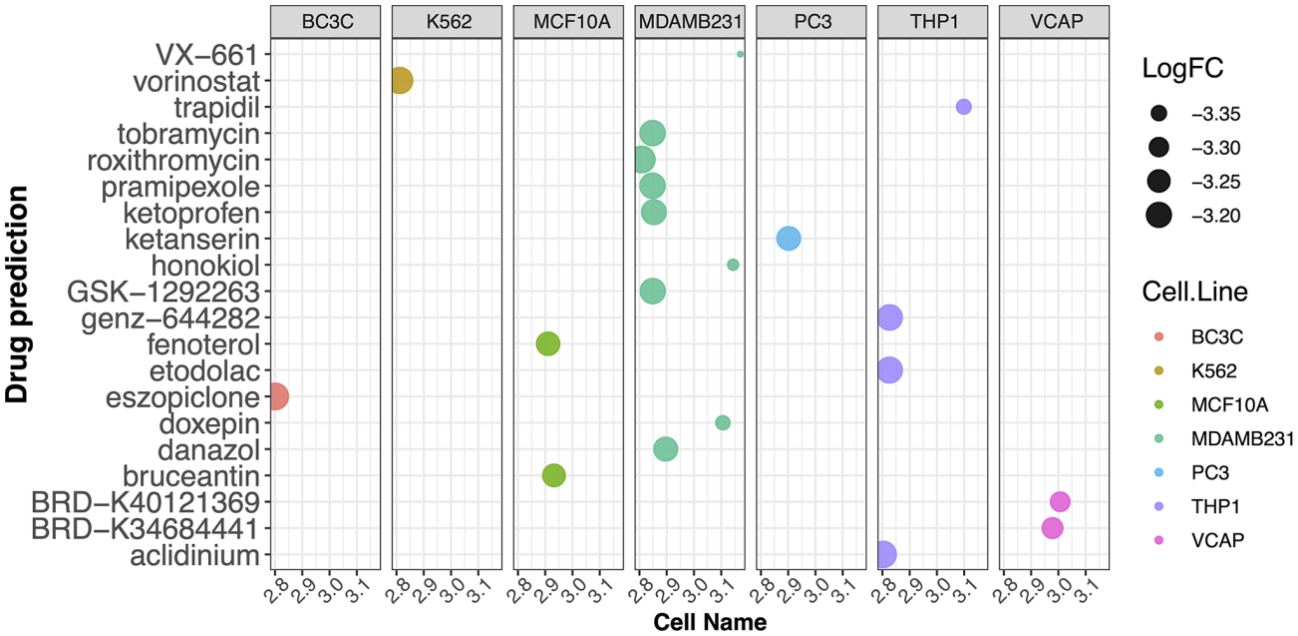
**Prediction of drugs targeting the potential diagnostic biomarkers using SigCom LINCS.** Top 20 small molecules related to expression regulation that are enriched for the four potential biomarker genes (z-score >3, *p*-value < 0.05) in seven cell lines.

Next, to investigate the potential mechanisms underlying the contribution of the four potential diagnostic biomarker genes to the occurrence of PD, we utilized the GeneMANIA online tool to construct a PPI network for these genes. The result is shown in Figure 4. As vividly shown in the figure, BRCA1 had a strong physical interaction with EGFR, which is crucial for susceptibility to PD in the Han Chinese population [26]. Furthermore, BRCA1 was predicted to have a significant association with PARD1 and TP53BP1. Pearson analysis was used to carry out the GSEA enrichment difference analysis in the human KEGG biological pathway set. The analysis revealed positive associations between the four potential diagnostic biomarker genes and the extracellular matrix (ECM)-receptor interaction and focal adhesion KEGG pathways. However, the analysis demonstrated negative associations between the four genes and the tricarboxylic acid cycle and ribosome KEGG pathways, as illustrated in Figure 5. Furthermore, we utilized SigCom LINCS to predict drugs targeting these biomarkers. The top 20 small molecules related to expression regulation enriched for the four potential diagnostic biomarker genes (z-score >3, *p*-value < 0.05) in seven cell lines are shown in Figure 6.

### Construction and evaluation of a nomogram model for PD diagnosis

**Figure 7:**
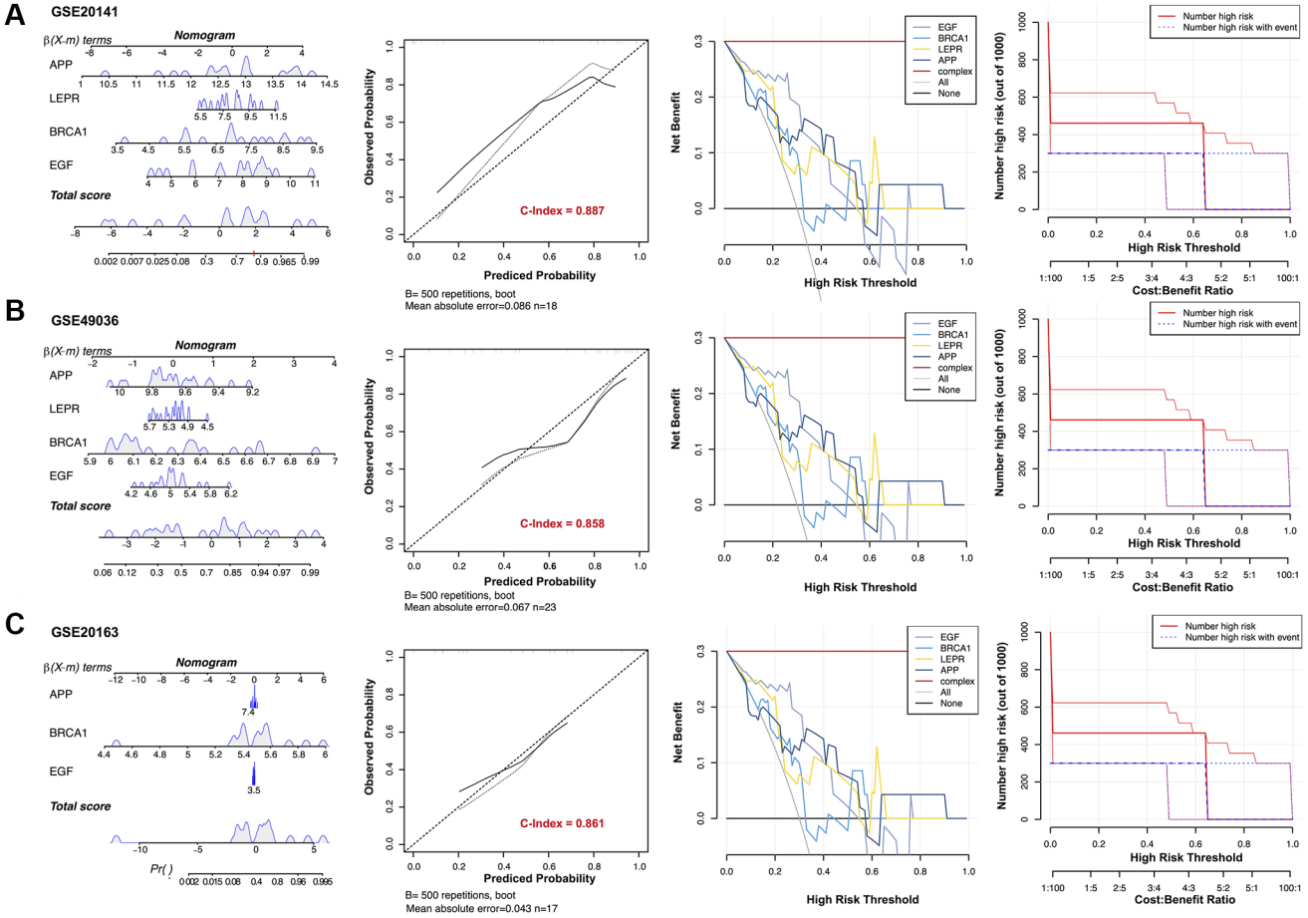
**Establishment of the diagnostic model in three validation datasets.** Nomograms for the diagnostic model of Parkinson’s disease (PD), calibration curves, and decision curve analyses (DCAs) for the diagnostic model were constructed in (**A**) GSE20141, (**B**) GSE49036, and (**C**) GSE20163.

**Figure 8:**
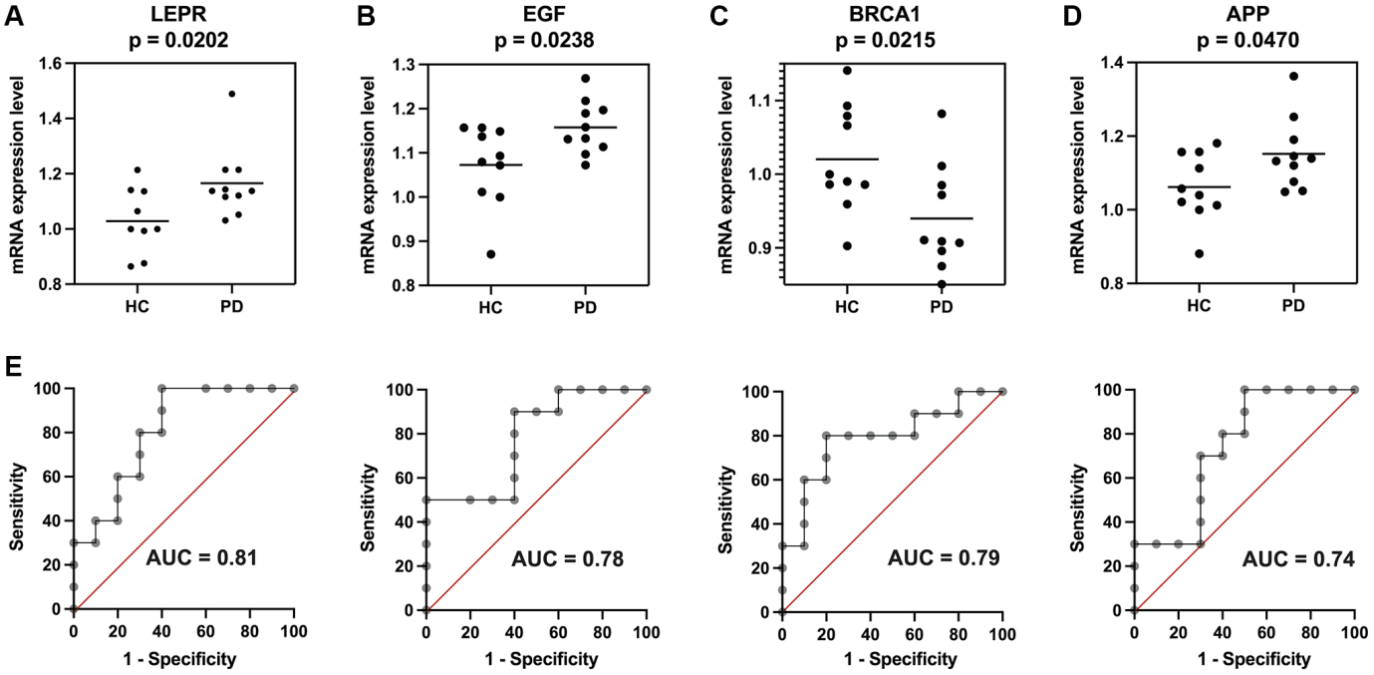
**Validation of the four potential biomarkers in an independent cohort.** Expression of (**A**) LEPR, (**B**) EGF, (**C**) BRCA1, and (**D**) APP in Parkinson’s disease (PD) patients and healthy control (HC) subjects. (**E**) Receiver operating characteristic (ROC) curves of the four genes for diagnosis. ^*^p < 0.05.

The Rms R package was utilized to develop a nomogram model for PD diagnosis, basing upon four genes (EGF, APP, BRCA1, and LEPR) in three validation datasets, as illustrated in Figure 7. Subsequently, a calibration curve was employed to assess the predictive performance of the nomogram model. The calibration curve revealed minimal discrepancy between the actual PD risk and the predicted risk, indicating high accuracy in PD prediction by the nomogram model, with a C-index of 0.887, 0.858, and 0.861 in GSE20141, GSE49036, and GSE20163, respectively (Figure 7). The Decision curve analysis (DCA) depicted that the nomogram curve surpassed the gray line as well as the individual curves for EGF, BRCA1, LEPR, and APP. This indicates that patients could derive greater benefit from the nomogram model across a high-risk threshold ranging from 0 to 1, exhibiting its superior clinical utility compared to individual gene-based models in all three validation datasets (Figure 7). Furthermore, to provide a more visual evaluation of the clinical impact, a clinical impact curve was plotted based on the DCA curve. The “Number high risk” curve closely aligned with the “Number high risk with event” curve across a high-risk threshold from 0.3 to 1, signifying the nomogram model’s exceptional predictive power (Figure 7). These findings suggest that the four potential diagnostic biomarker genes may indeed play a crucial role in the pathogenesis of PD. To further investigate their potential reliability as diagnostic biomarkers, expression levels of four genes were assessed via qRT-PCR in an independent cohort consisting of 10 PD patients and 10 HC subjects. The main clinical characteristics of both groups are summarized in Table 1. The results revealed that compared to the HC group, PD patients exhibited significantly higher expression levels of EGF, LEPR, and APP in their PBMCs (Figure 8A, 8B, 8D). However, BRCA1 was markedly decreased in PD patients (Figure 8C). ROC curve analysis showed that the AUC for EGF, LEPR, APP, and BRCA1 was 0.810, 0.780, 0.790, and 0.740, respectively, when distinguishing PD patients from HC subjects (Figure 8E).

**Table 1:** Demographic and cognitive assessment scores of participants.

**Characteristic**	**HC, *n* = 10**	**PD, *n* = 10**	***P***
Age, yr	61.2 ± 12.6	56.7 ± 11.4	0.185
Gender, M/F	43/49	54/38	
Education, yr	13.51 ± 6.72	11.93 ± 5.84	0.089
MoCA, mean ± SD (range)	26.1 ± 1.3 (25–30)	25.2 ± 1.7 (24–30)	0.081
BDI	4 ± 3	6 ± 3	0.332
LEDD, mg	—	484.87 ± 310.93	
PD duration, yr	—	4.40 ± 3.59	
H&Y scores	—	2.1 ± 0.7	
UPDRS III	—	25 ± 8	

Abbreviations: BDI: Beck Depression Inventory; F: female; LEDD: mean L-dopa equivalent daily dose; M: male; MoCA: Montreal Cognitive Assessment; PD: Parkinson disease patients; UPDRS III: Unified Parkinson Disease Rating Scale, part III (motor).

### Correlation between the two biomarkers and immune cell infiltration

**Figure 9:**
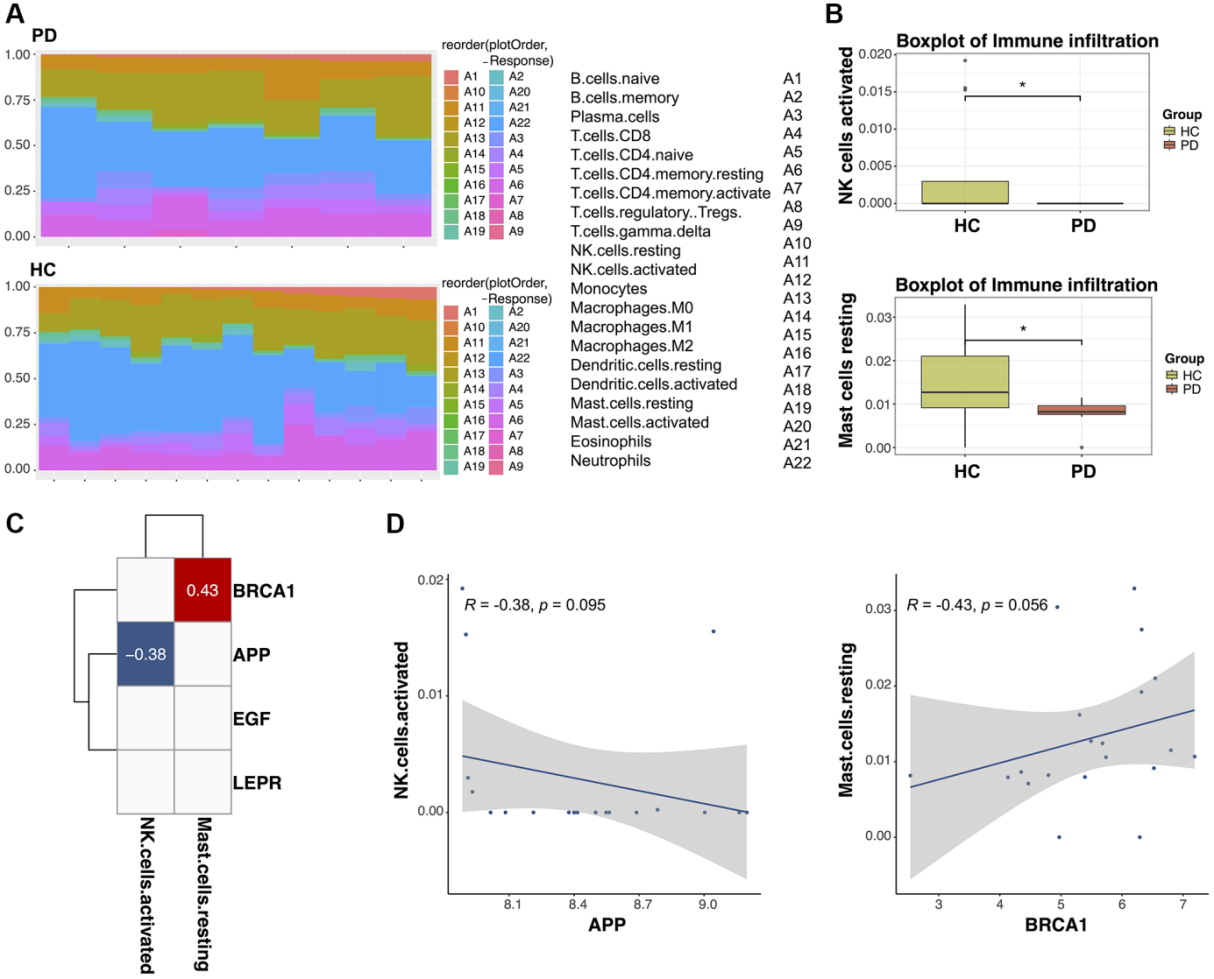
**The relationship between diagnostic biomarkers and immune cell infiltration.** (**A**) Heatmap of the infiltration proportions of 22 types of immune cells in Parkinson’s disease (PD) patients and healthy control (HC) subjects. (**B**) Box plots for the differential proportion analysis of two immune cell types in PD and HC samples. (**C**) Heatmap of the correlation between diagnostic biomarkers and immune cells. (**D**) Correlation between APP expression and activated natural killer (NK) cells and correlation between BRCA1 expression and resting mast cells.

There is substantial evidence suggesting that dysfunction of the immune system contributes to Parkinson’s disease (PD). This evidence comprises various investigation, including clinical and genetic links between autoimmune disease and PD, impaired immune responses at the cellular and humoral levels in PD patients, imaging data revealing activation of inflammatory cells in affected brain regions, and observations of immune dysregulation in PD animal models [27, 28]. Therefore, we compared immune infiltration in PD patients and HC subjects. The ssGSEA algorithm was employed to assess the infiltration of 22 immune cell types in each sample, with the results visualized in a heatmap (Figure 9A). Subsequently, the Wilcoxon test was applied to compare the expression levels of immune cells between PD patients and HC subjects. Notably, in Figure 9B, it is evident that resting mast cells and activated natural killer (NK) cells exhibited reduced expression in PD patients compared to HC subjects (*p* < 0.05). Furthermore, a correlation analysis revealed associations between the expression levels of two biomarker genes (BRCA1 and APP) and the presence of the two immune cell types (*p* < 0.1, |Coef| > 0.2). Specifically, APP showed a negative correlation with active NK cells (r = −0.38, *p* = 0.095), whereas BRCA1 demonstrated a positive correlation with resting mast cells (r = 0.43, *p* = 0.056; Figure 9C, 9D). These findings suggest a potential regulatory role of these biomarkers in PD through interactions with the immune microenvironment.

## DISCUSSION

In this study, RNA-Seq data from 11 PD patients and 13 HC subjects were analyzed using multiple bioinformatic approaches. A total of 593 downregulated genes and 842 upregulated genes with differential expression between PD and HC were subjected to GO and KEGG analyses to elucidate their functional implications. GO analysis found that downregulated DEGs are enriched in the neutrophil activation and neutrophil-mediated immunity terms, indicating a potential involvement of inflammation in PD pathology. The inflammatory mechanisms implicated may include: (1) Abnormal aggregation of α-synuclein, leading to conformational changes that activate toll-like receptor 4 and subsequently trigger the release of numerous proinflammatory cytokines. This cascade of events contributes to the pathogenesis of PD, (2) chronic peripheral inflammation and immune activation result in elevated levels of proinflammatory cytokines in PD patients. These cytokines may enter the brain through the traditional endocrine route or via direct transmission through the vagus nerve. Elevated cytokine levels can lead to disruption of the blood-brain barrier (BBB) by interacting with endothelial cells, facilitating the passage of proinflammatory cytokines across the BBB and into the brain. This process further promotes the development of PD [27]. Although KEGG pathway analysis indicates that DEGs are implicated in the developmental pathway of measles, substantial evidence suggests that many childhoods viral infections are correlated with a decreased risk of PD [29]. However, our KEGG analysis results revealed that the upregulated DEGs were notably enriched in pathways such as ribosome assembly, PD, and oxidative phosphorylation. In the pathological progress of PD, the abnormal expression of signaling pathways, metabolic reprogramming, and noncoding RNA can promote the transcriptional activity of RNA polymerase I, resulting in the over-activation of ribosomes [30]. Moreover, reactive oxygen species (ROS) play a pivotal role in PD development, leading to significant damage and death of dopamine cells. ROS are generated through various intracellular mechanisms, such as NADPH oxidase activation, mitochondrial dysfunction, and hydrogen peroxide (H_2_O_2_) breakdown [31].

Through four machine learning algorithms, we found aging-related DEGs, including BRCA1, CHEK2, CLU, APP, LEPR, and EGF, that served as robust diagnostic biomarkers. Then, using LASSO regression, we reduced dimensions and pinpointed four genes (EGF, BRCA1, LEPR, and APP) to create a diagnostic model for PD. Across various datasets, all four genes exhibited robust diagnostic efficacy, suggesting their potential as biomarkers for diagnosing PD. Notably, EGF’s expression serves as a neurotrophic factor in nigrostriatal dopaminergic neurons [32, 33]. It enhances tyrosine hydroxylase expression, boosts dopaminergic turnover in the striatum, and prevents dopaminergic neuron degeneration [34]. Interestingly, the combination of IGF-1 and EGF in serum is found to be more useful for PD diagnosis than EGF alone. It is reported that EGF promote the survival and regeneration of dopaminergic neurons in the substantia nigra [34], the primary type of neuron lost in PD. The neuroprotective effects of EGF could be through activation of intracellular signaling pathways that inhibit apoptosis (programmed cell death) and promote cell survival, which might be potential mechanism of EGF in prodromal stage of PD. Mounting evidence indicates that BRCA1 plays a pivotal role in DNA damage and apoptosis induced by oxidative stress [35]. In mice with BRCA1 loss-of-function mutations, there is a notable increase in ROS levels [36, 37]. BRCA1’s main function is in DNA repair, specifically in the repair of double strand breaks through homologous recombination [35]. In neurodegenerative diseases like PD, DNA damage in neurons can accumulate over time, contributing to cell death and disease progression. BRCA1 might influence PD pathogenesis through its role in maintaining genomic integrity in neurons, potentially affecting the cells’ vulnerability to degeneration.

The protein leptin, encoded by the LEPR gene, acts as a key regulator of energy balance and feeding behavior, while also demonstrating neurotrophic effects during both perinatal central nervous system development and into adulthood [38]. Research by Ho et al. has revealed that leptin plays a protective role in neuronal SH-SY5Y cells against MPP+ toxicity, by sustaining ATP levels and mitochondrial membrane potential [39]. The observed upregulation of the LEPR gene suggests a potential activated feedback mechanism by neurons for protection, a hypothesis that warrants further investigation, particularly in larger PD cohorts [38]. LEPR mediates the actions of leptin, a hormone involved in regulating energy balance [39], but also implicated in inflammation and immunity [40]. In PD, inflammation is a key component of the disease’s progression, with activated microglia and astrocytes contributing to neuronal damage. Leptin, through LEPR, could modulate neuroinflammation, potentially exerting neuroprotective effects by reducing inflammatory responses in the brain or influencing energy metabolism in neurons, thereby affecting their survival, which might be potential mechanism of LEPR in pathology of PD. Existing data suggest that abnormalities in the metabolism of APP can contribute to mitochondrial dysfunction, leading to cell death [41]. Overexpression of APP can result in elevated levels of its metabolite, Aβ peptide, which can reach toxic levels. The toxicity of Aβ leads to the generation of ROS, induction of apoptosis, and impaired memory [42]. APP has been a focal point of research due to its potential role in causing mitochondrial dysfunction [43]. Additionally, APP is linked to dysfunctions in autophagy and apoptosis in neurodegenerative disorders [43]. In PD, while the primary focus has been on α-synuclein, there’s evidence that Aβ accumulation might also play a role in the PD pathophysiology [44], potentially through similar mechanisms of neurotoxicity, such as oxidative stress, mitochondrial dysfunction, and synaptic loss [43]. APP’s processing and the subsequent accumulation of Aβ could therefore be a shared pathological feature in PD and Alzheimer’s disease.

Our study measured RNA expression levels in whole blood, so the study better reflects the usefulness of the four potential biomarker genes in PD diagnosis. The results of qRT-PCR in our study revealed that APP, EGF, and LEPR were significantly upregulated and that BRCA1 was significantly downregulated in PD patients relative to HC subjects, indicating that the four aging-related genes might be reliable diagnostic biomarkers.

When comparing potential molecular biomarkers for Parkinson’s Disease (PD) like EGF, BRCA1, LEPR, and APP with recognized biomarkers, it’s essential to understand the context of their roles and advantages in PD. The recognized biomarkers for PD primarily involve α-synuclein, LRRK2 gene mutations, and others that directly relate to the pathogenesis of PD. The new above potential candidates are implicated in a diverse range of biological processes and could offer new insights or advantages in understanding, diagnosing, or treating PD. EGF has been implicated in neurogenesis and neural repair. It can play a role in protecting dopaminergic neurons from degeneration [34], which is a hallmark of PD. If proven to be a reliable biomarker, EGF could serve as a tool for assessing the efficacy of therapies designed to enhance neuronal repair and survival. Additionally, it may offer insights into disease progression related to neuronal loss. While BRCA1 is well-known for its role in hereditary breast and ovarian cancer, there’s emerging evidence that it may also play a role in neuronal health, particularly in DNA repair mechanisms [35]. BRCA1 as a biomarker could indicate the integrity of DNA repair mechanisms in neurons, offering a novel therapeutic target or diagnostic criterion focusing on genomic stability in PD. Leptin is involved in energy homeostasis and has been shown to have neuroprotective effects. The leptin receptor’s role in PD could relate to metabolic changes or inflammation associated with the disease [40]. LEPR could offer insights into the metabolic and inflammatory aspects of PD, potentially leading to biomarkers that predict disease progression or response to therapies addressing these aspects. APP is famously associated with Alzheimer’s disease, but its role in PD might relate to Aβ accumulation and its effects on neuronal health [44]. Understanding APP’s role in PD could lead to biomarkers for neurodegenerative processes that overlap with other conditions like Alzheimer’s, offering a broader approach to diagnosing and treating neurodegeneration. Compared to recognized PD biomarkers focused on the disease’s core pathophysiological features (e.g., α-synuclein aggregation), these candidates could provide a broader understanding of PD’s impact on neuronal health [45], metabolism [46], DNA repair [47], and amyloid processing [48]. They might offer new avenues for therapeutic intervention, early detection, and monitoring of disease progression by targeting different aspects of the disease’s pathology. However, the utility of these potential biomarkers in PD still requires substantial research and validation to determine their effectiveness and advantages over existing markers.

Through GSEA analysis, we discovered that the four potential diagnostic biomarker genes exhibit positive correlations with the ECM-receptor interaction pathway and the PI3K-AKT signaling pathway, while showing negative correlations with the Th1 and Th2 cell differentiation pathways [49]. ECM molecules, along with their cell surface receptors and adhesion molecules, are associated with a range of central nervous system disorders, such as Alzheimer’s disease, schizophrenia, epilepsy, multiple sclerosis, addiction, and PD [49]. The ECM-receptor interaction and PI3K-AKT signaling pathways [49], known regulators of autophagy, may play a pathogenic role in PD [50]. JKAP inactivation of T-cell signaling and interaction with Th1 and Th17 cells result in a dysregulated immune environment and inflammation, impacting PD progression [51]. Consequently, it correlates with certain portions of MMSE and UPDRS scores [51]. Several studies have implicated Th1 and Th2 cells in PD [52–54].

The immune-inflammatory response and regulation of immune cells are implicated in PD pathogenesis. In a training dataset comprising 24 samples, we observed significant differences in activated NK cells and resting mast cells between PD patients and HC subjects. NK cells have been found to infiltrate the SN of PD mice, where they colocalize with α-synuclein and dopaminergic neurons. Moreover, the ratio of NK cells to other immune cells is increased in the CSF of PD patients [55]. Neurovascular PD patients exhibit alterations that not only trigger innate immune responses but also attract and activate adaptive responses [56, 57]. Increased levels of cytokines such as IL-1β, IL-2, IL-6, IFN-γ, and TNF-α, along with elevated CD4+ lymphocyte counts, have been observed in both the serum and CSF of individuals with PD. Additionally, research on immune infiltration has identified associations between regulatory T-cells, monocytes, and resting mast cells with PD pathogenesis [58].

Our research offers several advantages. Firstly, we pioneered the development of an aging-related diagnostic signature for PD and confirmed its strong association with PD patients in our RNA-Seq dataset. Secondly, the diagnostic biomarkers we identified exhibited high efficiency across various datasets. However, our study is subject to certain limitations. The training dataset had a limited sample size, comprising only 24 samples. Additionally, further experiments are necessary to validate our findings, including validation in larger sample size and more rigorous designed trials, as well as assessing the expression of the four potential biomarker genes in other neurodegenerative diseases.

## MATERIALS AND METHODS

### Collection of clinical characteristics

The study sample consisted of two groups: PD patients (*n* = 11, seven men, four women; mean age 62.33 ± 10.49 years) and healthy control (HC) subjects (*n* = 13, eight men, five women; mean age 63.56 ± 9.26 years). All participants were sourced from the outpatient clinic for movement disorders at Xuanwu Hospital, Capital Medical University. Clinical assessments, including PD diagnosis and staging, were conducted by experienced neurologists. PD patients were receiving pharmaceutical treatment. HC subjects were selected from the Bank of Aged Healthy Population in Xicheng District, Beijing. The study received approval from the ethics committee of Xuanwu Hospital, Capital Medical University, and all participants provided their consent in writing after being informed about the study.

### Data sources

Aging-related genes, totaling 307, were extracted from the Human Ageing Gene Database available at http://genomics.senescence.info/genes/ for inclusion in this study. A volume of 400 μL of peripheral blood was collected from 11 PD patients and 13 HC subjects in PAXgene Blood RNA tubes (PreAnalytiX) and frozen at −80°C for long-term storage as described by Wylezinski et al. [59]. RNA was extracted from the peripheral blood and preserved in PAXgene Blood RNA tubes in accordance with the manufacturer’s instructions, utilizing an automated QIAcube system (Qiagen). Following this, RNA samples underwent purification, concentration, and elution in RNase-free water, utilizing the RNEasy MinElute Cleanup Kit (Cat. No. 74204, Qiagen).

The Illumina NovaSeq 6000 RNA sequencing platform was used in the PE150 sequencing mode. We employed Rsem software (Version 1.2.6), which utilizes the FPKM (Fragments Per Kilobase per Million mapped reads) method, to calculate gene expression levels for mRNA [60].

The RNA-Seq data, including 11 PD samples and 13 HC samples, were used as a training dataset for analysis for follow-up research. The expression profiles of 10 PD and 8 HC individuals from GSE20141, 8 PD and 9 HC individuals from GSE20163, and 15 PD and 8 HC individuals from GSE49036 were obtained from the Gene Expression Omnibus (GEO) database at http://www.ncbi.nlm.nih.gov/geo/. Self-test RNA-Seq was employed to pinpoint potential PD diagnostic biomarkers, while the three expression profiles from GEO, containing 33 PD and 25 HC samples, were combined to assess the accuracy of these biomarkers. RNA-Seq data underwent processing, including normalization of count reads from preprocessed data (involving transcript abundance estimation and sequence alignment). Subsequently, a log2 transformation was conducted, with the addition of a 0.5 pseudocount.

### DEGs identification and functional analysis

Differentially expressed genes (DEGs) between PD and HC in the self-test RNA-Seq data were identified using the limma R package, with criteria of |LogFC| >1 and *p*-value < 0.05 [61]. Using the ggplot2 R package, volcano plots depicted the DEGs, while the heatmap illustrated the expression levels of these genes. The RobustRankAggreg R package facilitated the accurate identification of DEGs between PD and HC. Functional analysis of the DEGs was conducted using the clusterProfiler R package. Significance was determined at a *p*-value < 0.05.

### Machine learning

Genes present in both the DEGs and the 307 aging-related genes were identified and represented in a Venn diagram. Following the identification of shared genes, STRING [62] was utilized to acquire protein-protein interactions (PPIs) with a minimum required interaction score of 0.4 (medium confidence). Subsequently, the network was visualized using Cytoscape V3.9.1 software [63].

Machine learning techniques, such as LASSO, SVM, RF, RR, were implemented utilizing the glmnet, randomForest, e1071, and glmnet R packages, respectively, to construct classifiers of diagnosis. Six potential diagnostic biomarkers were identified using the four machine learning methods and visualized in a Venn diagram.

LASSO regression was employed to identify the optimal combination of genes for constructing a diagnostic signature. Subsequently, a multivariate Cox regression model was utilized to further refine the identified genes, employing the R “step” function.

### Diagnostic biomarkers identification and drugs prediction

ROC curve analysis assessed the efficacy of aging-related DEGs in distinguishing PD from HC, identifying DEGs with an AUC exceeding 0.7 as potential PD diagnostic biomarkers. Validation in datasets GSE20163, GSE49036, and GSE20141 confirmed their reliability. SigCom LINCS [64] (https://maayanlab.cloud/sigcom-lincs/) was employed to identify drugs (small molecules) targeting above genes. LINCS L1000 Chemical Perturbations (2021) was screened as the prediction result of small molecules, and the small molecules with a *p*-value < 0.05 and z-score >3 were screened for, constructed, and visualized [65].

### Analysis of diagnostic genes by PPI and GSEA

After identifying the potential diagnostic biomarker genes by using the four machine learning algorithms, the GeneMANIA database (https://genemania.org) was used to analyze them and their 20 interacting genes based on PPIs. Subsequently, the gene-gene network was generated and visually represented.

Pearson analysis was employed to measure the gene set enrichment analysis (GSEA) difference of PD diagnostic genes in the human KEGG biological pathway set. GSEA was conducted utilizing GSEA software (v 4.0, https://www.gsea-msigdb.org/gsea/index.jsp) on the training dataset (24 samples).

### Development and validation of a nomogram model for diagnosing PD

The rms R package was utilized to construct a nomogram model predicting PD occurrence across three validation datasets (GSE20141, GSE49036, and GSE20163). Each factor’s score is represented by “Points”, and the cumulative score of all factors is denoted by “Total Points.” The predictive performance of the nomogram model was evaluated using the C-Index.

### Immuno-relevance analysis of potential diagnostic biomarker genes

The proportions and distributions of immune cells were quantified using the CIBERSORT [66] algorithm, based on RNA-Seq data from the training dataset. The abundances of 22 types of immune cells were calculated using the LM22 signature algorithm. After calculating the proportion of immune infiltrative cells, the Mann-Whitney *U*-test was employed to assess and analyze the differences between the immune infiltration status of PD patients and HC subjects (*p*-value < 0.05) to screen for PD-related immune infiltration states. After determining the immune state that was significantly associated with PD, we investigated the correlation between potential biomarker genes and immune infiltration cells (*p* < 0.05, |Coeff| > 0.2) in accordance with Spearman correlation analysis.

### Extraction of RNA followed by qRT-PCR

Blood samples were collected in PAXgene Blood RNA tubes, followed by isolation of peripheral blood mononuclear cells (PBMCs) using density gradient centrifugation (Solarbio Life Science, Beijing, China). Total cellular RNA extraction from the PBMCs was carried out using the RNA Extraction Kit (Omega, Guangzhou, China). Subsequently, 200 ng of RNA per sample underwent reverse transcription using the Evo M-MLV RT Kit (Accurate Biotechnology, Changsha, China) following the manufacturer’s instructions. qRT-PCR was conducted using the SYBR Green Premix Pro Taq HS qPCR Kit (Accurate Biotechnology, Changsha, China) on the Light Cycler 40 real-time PCR instrument (Roche, Basel, Switzerland). The relative expression of mRNA was calculated using the 2^−ΔΔCt^ method with GAPDH as the internal control for normalization. The following primers were employed: LEPR-forward 5′-GGGAAGATGTTCCGAACCCCA-3′, EGF-forward 5′-TTCACTGTCTTGACTCTACTCCACC-3′, BRCA1-forward 5′-TGAGAAGCGTGCAGCTGAGA-3′, APP-forward 5′-TGGTTCGAGTTCCTACAACAGCA-3′, and LEPR-reverse 5′-AGGACCACATGTCACTGATGCT-3′, EGF-reverse 5′-CATCGCTCCCCGATGTAGCC-3′, BRCA1-reverse 5′-TGTCACTCTGAGAGGATAGCCC-3′, APP-reverse 5′-TGACGTTCTGCCTCTTCCCA-3′. Four mRNA primers were designed using tailing methods. All experiments were conducted in triplicate.

### Statistical analysis

All data analyses were carried out using R 4.0.3 software, SPSS 26.0, and GraphPad Prism 8. Pearson’s correlation test was utilized for correlation analysis. The Wilcoxon test and the Mann-Whitney *U*-test were employed for comparing two groups, while the Kruskal-Wallis test was used for comparing more than two groups. A significance level of *p* < 0.05 (two-tailed) was applied for determining statistical significance.
